# Antenatal care utilization and its associated factors in Somalia: a cross-sectional study

**DOI:** 10.1186/s12884-023-05871-4

**Published:** 2023-08-12

**Authors:** Alli Miikkulainen, Ibrahim Abdirahman Mohamud, Majda Aqazouz, Bishara Abdullahi Suleiman, Omar Sheikh Mohamud, Abdifatah Ahmed Mohamed, Rodolfo Rossi

**Affiliations:** 1 International Committee of the Red Cross Somalia, Nairobi, Kenya; 2 International Committee of the Red Cross Somalia, Mogadishu, Somalia; 3 International Committee of the Red Cross Regional, Nairobi, Kenya; 4 Somali Red Crescent Society, Mogadishu, Somalia; 5 Federal Ministry of Health, Mogadishu, Somalia; 6 International Committee of the Red Cross, Geneva, Switzerland

**Keywords:** Antenatal care, Pregnancy, Breastfeeding, Health facility delivery, Somalia

## Abstract

**Background:**

WHO recommends attending minimum four ANC consultations during pregnancy to ensure early detection of complications. The objective of this study was to quantify ANC attendance and factors associated with it.

**Methods:**

Participants were randomly selected using the WHO Cluster survey methodology in Southern and Central Somalia. A paper-print questionnaire was used to collect all data. Outcomes of interest were: access to at least one ANC consultation, completion of at least four ANC consultations, initiation of breastfeeding and place of delivery, while exposures included factors related to the latest pregnancy and demographic characteristics. Associations were assessed through logistic regression.

**Results:**

Seven hundred ninety-two women answered the questionnaire; 85% attended at least one and 23% at least four ANC consultations, 95% started breastfeeding and 51% had an institutional delivery. Encouragement to attend ANC increased the odds of attending at least one consultation (aOR = 8.22, 95%CI 4.36–15.49), while negative attitude of husband or family decreased the odds (aOR = 0.33, 95%CI 0.16–0.69). Knowing there is a midwife increased the odds of at least four visits (aOR = 1.87, 95%CI 1.03–3.41). Attending at least four consultations increased the odds of delivering in a health structure (aOR = 1.50, 95%CI 1.01–2.24), and attending at least one consultation was associated with higher odds of initiating breastfeeding (aOR = 2.69, 95%CI 1.07–6.74).

**Conclusions:**

Family has a strong influence in women’s ANC attendance, which increases the likelihood of institutional delivery and initiating breastfeeding. Women and families need to have access to information about benefits and availability of services; potential solutions can include health education and outreach interventions.

## Background

Around the world, every day about 810 women die from preventable causes related to pregnancy and childbirth, with considerable disparity in the geographical repartition where about 94% of all maternal deaths occurring in low and middle income countries [1]. Many of those complications occurring during pregnancy, childbirth and post-partum are preventable or treatable [2]; in some cases complications might exist prior to the pregnancy, yet the gestation can exacerbate women and baby’s health condition mainly if not managed in a timely manner. Main major complications leading to maternal deaths worldwide, and representing almost 75% of maternal death, are severe bleeding (mostly bleeding after childbirth), infections, high blood pressure during pregnancy (pre-eclampsia and eclampsia), complications from delivery and unsafe abortion [3].

Preventive interventions during pregnancy are recommended for healthy positive pregnancy experience and early detection of complications [4]. Among those models of care intending to prevent and detect complications in pregnancy we mention Antenatal care (ANC), which refers to the care and attention provided by trained health professionals to women throughout their pregnancies. It includes individual relation between health care provider and pregnant women, follow up of physical and mental status, focusing on screening and risk identification, prevention and management of pregnancy-related and co-occurring conditions, beside health education and promotion [5]. Early identification of complications revealed during ANC is an essential factor to promptly be managed or stabilized at Primary Health Care or refer pregnant women to higher levels of care where they can adequately address the condition with appropriated care. The International Confederation of Midwives (ICM) identifies midwife as the profession of choice for the care of childbearing women [6].

The World Health Organization (WHO) recommends starting ANC consultations in the first trimester of pregnancy and making at least four consultations (ideally eight) during pregnancy. Specifically, WHO, in its ANC guidelines, advises pregnant women to make their first contact during the first 8–12 weeks of gestation, followed by the second visit between 24–26 weeks, the third visit at 32 weeks, and the forth visit between 36–38 weeks of gestation [7].

Attendance to ANC consultation is an essential component in maternal health [8]. Nevertheless, challenges are still blocking not only the early initiation of ANC, but also the follow up consultations, including proper preparation of individual birth plans [9].

Globally, while 86% of pregnant women have access to at least one antenatal care visit by a skilled health worker, only 65% of women who have access to ANC receive at least four antenatal visits. In regions with the highest maternal mortality rates, such as sub-Saharan Africa, as few women as 52% receive at least four antenatal visits [10]. This percentage can be lower in humanitarian contexts, with 21% in Afghanistan, 25% in Somalia, and 38% in Niger [11].

The total population of Somalia is estimated to be 17,066,000, including 3,758,000 women in reproductive age [12], with an average fertility rate of 6.9 children per woman [13].

Women in Somalia have one in 22 lifetime risk of maternal death making its maternal mortality rate (MMR) amongst the highest in the world, estimated to be at 692 deaths per 100,000 live births according to the Somali Health and Demographic Survey done in 2020. The main causes of maternal mortality identified include both antepartum and postpartum hemorrhage (24.8%), preeclampsia/eclampsia (12.9%), obstructed labour (6.9%), unsafe abortion (12.9%) and sepsis (14.9%). Common causes of indirect obstetric death, which account for 19.8% of the maternal deaths, include exacerbation of cardiac or renal disease, severe anemia, Tuberculosis, violence related to pregnancy state and domestic violence, homicide, suicide and HIV disease [13]. The Voluntary National Review Report published in 2022 identified several reasons for high maternal mortality rate in Somalia, amongst them low uptake of antenatal and postnatal care, and a low number of deliveries at health facilities or with skilled health care providers [14]. As a result of chronic conflict and related reasons such as poverty and lack of education opportunities, Somalia is facing a chronic shortage of midwives [15]. To bridge this gap, trained female community health workers (FCHW) have a crucial role at the community level in providing health education and referring women to skilled health care providers for ANC. While midwives encourage women to return to the health facility for delivery, clean delivery kits are also provided during the last trimester to ensure hygienic home delivery [16].

According to the Somali Health and Demographic Survey carried out in 2020, 31% of women aged 15–49 who had a live birth received ANC from skilled personnel during their last birth and 24% of the women had at least 4 ANC visits [13], while only 17% of women received two or more tetanus toxoid injections during the pregnancy of their last live birth [17]. An overwhelming 89% of the mothers did not receive any postnatal check-up in the first two days after childbirth [13].

According to the same report, approximately 32% of deliveries were reported to be assisted by skilled health attendant including doctors/clinical officers, nurses and midwives, while the remaining 68% were reported either non assisted deliveries or deliveries assisted by non-skilled attendant [13]. Regarding places of delivery, it was found that 79% of deliveries took place at home, mostly with the assistance of traditional birth attendants, and only around one in five births (21%) occurred in a health facility [18].

Routinely collected data at the Primary Health Care level by the Somali Red Crescent Society (SRCS) and the International Committee of the Red Cross (ICRC) for 2021 showed that ANC1 coverage was virtually 100%, while the coverage for ANC4 was about 88% (internal data, not published). However, there may always be discrepancies between the data collected for routine monitoring and those collected on the purpose of research.

While ANC guidelines are systematically updated by WHO and recommended to all Member States, other challenges might impede the effectiveness of focused ANC with at least 4 ANC consultations per pregnant women.

In order to understand some aspects of those challenges, it is important to assess what are the factors associated to access and beliefs around ANC among Somali women who recently delivered. More specifically, the objectives of the study are:

- Assessing a more precise estimation for ANC coverage and therefore validate what is seen in routine monitoring through ICRC and SRCS Database,
- Exploring factors that are associated with access to at least 1 and the full cycle of 4 + ANC consultations,
- Assessing whether access to ANC increases the likelihood of delivery in a health structure and (at least initiation) breastfeeding of the newborn.

## Methods

### Study design and study population

We conducted an analytical community-based cross-sectional study to be representative of all regions of Southern and Central Somalia, in the catchment areas of SRCS primary health care clinics, which cover the whole South-Central Somalia due to the presence of at least one SRCS clinic in each district. The study population consisted of women of at least 15 years of age, who had delivered within the last 12 months, regardless of the outcome of the delivery (live or stillbirth). Women were retrospectively interviewed about ANC attendance and other factors. Girls younger than 15 years or women who had never delivered or delivered over 12 months before the survey were excluded from the study.

### Main outcomes and other variables

The main outcomes were the following:

- Access to at least one antenatal care (ANC) consultation
- Completion of at least four ANC consultations
- Initiation of breastfeeding of the last-born child
- Place of delivery (health institution vs. community delivery)

Other variables (as possible exposures) included various demographic (e.g. age, parity, living arrangements) and socioeconomic variables (measured as proxy from education and occupation, since monetary income was considered as a sensitive question to ask), access and availability related variables (e.g. distance from health facility, availability of midwife, availability of services), and variables related to behavior, practices and beliefs around ANC (e.g. encouragement from others, knowledge about complications, knowledge about benefits of ANC).

For women who had not attended the full ANC cycle, follow-up questions were asked about the reasons. Similarly, follow-up questions were asked about knowledge and experience of complications, encouragement to attend ANC and reasons to choose the place to give birth. For some questions, respondents could select more than one answer.

### Sample size calculation and selection of clusters

The required sample size to allow a ± 5% confidence limit, a power of 80% and alpha 0.05 was 800 women. We selected 40 clusters with 20 participants per cluster. Selection of clusters was done by using the cluster random sampling method, as developed by the WHO [19]. The probability for a cluster to be selected was proportional to the population size of each village. The list of villages with estimated populations was obtained from SRCS. Cumulative population was then calculated and divided by the number of clusters (forty) to obtain the sampling interval. A random number table was used to identify the first cluster. Identification of the subsequent clusters was obtained by adding each time the result for the sampling interval.

Known places (e.g. mosques, marketplaces, schools, monuments) were selected as starting point per each cluster. In each of these points, the survey team span a pen to randomly define the direction to find the 20 women.

### Questionnaire and data collection

Data was collected using a structured questionnaire that included 34 questions about the outcomes and exposures of interest. The questionnaire was first designed in English, translated into Somali, and independently, re-translated into English to check for consistency. The questionnaire was also tested with a small sample (10 women).

Data collection was done by 82 SRCS volunteers who were previously trained on data collection techniques (including how to minimize biased answers), confidentiality and consent, and supervised by senior SRCS and ICRC staff. Volunteers worked in pairs, and female volunteers were prioritized in order to increase the response rate and the possibility to conduct the interviews alone. Printed questionnaires were used for data collection. The principal investigators were involved in the training of volunteers, but not in the data collection.

### Data management and statistical analysis

Data entry in a Microsoft Excel sheet was done by a trained team of four data clerks and supervised by the research team, using pre-defined numerical codes. Each of the four data clerks first entered data for their respective clusters, after which the data was cross-checked by another data clerk. Questions with multiple choice option were entered into a separate sheet for descriptive analysis. Only the team involved in data cleaning and data analysis had access to the dataset.

Descriptive and analytical statistics were performed with Stata 16 software. Categorical variables were first explored by tabulation and reported as frequencies and percentages. Continuous variables, such as number of children and age, were explored according to the mean, median, standard deviation (SD) and range; then were categorized either by using the median as a cut-off point or according to other criteria.

Assessment of associations was done through univariable and multivariable logistic regression to report crude and adjusted odds ratios (ORs) accompanied by 95% confidence interval (95%CI) and *p*-value from Wald Test. We included in the multivariable models only variables that appeared to be statistically associated with the given outcome or that were considered as programmatically or clinically important. We considered as statistically significant *p*-value < 0.05 and marginally significant *p*-values included between 0.05 and 0.1.

### Ethical considerations

Ethical approvals were received from the Research & Ethical Committee at the Federal Ministry of Health & Human Services in Somalia on 14 February 2022 (MOH&HS/DGO/0185/February/2022), ICRC Ethics Review Board on 16 February 2022 (DP_DIR 21/00005), and SRCS Executive Director on 10 March 2022 (SRCS/MCO/261/2022).

Due to the high proportion of early marriage and pregnancy in Somalia, the study population included girls aged 15 years and older. All study participants or their accompanying relative provided informed verbal consent as follows: In case of interviewees younger than 18, an informed consent was collected from an accompanying relative, while the interview would still be done alone to avoid respondent bias. Upon advice of the local authorities, only verbal consent was requested due to high illiteracy rate in Somalia and to protect the identity of the respondent. No names were collected, therefore protecting the identity of participants, and potentially increasing the acceptability of the study. The ICRC Ethics Review Board accepted the approach of verbal informed consent as opposed to a written one.

The study was designed and conducted in accordance with the Declaration of Helsinki, as required for any study involving human participants.

## Results

### Cluster attribution and response rate

The random systematic sampling identified 40 clusters among the total list of 299 villages. 36 individual villages were attributed clusters, majority of which had 1 cluster. A few of the villages with a larger population ended up having up to 3 clusters.

In each cluster, the teams approached 20 eligible women to reach the required sample size of 800 women. 792 women accepted to answer the questionnaire, resulting in a response rate of 99%.

### Descriptive results

#### Demographic characteristics

Age of participants ranged between 16–48 years, with a mean of 27.75, standard deviation (SD) of 6.23 and median of 27 years. Total number of children the woman had given birth to ranged between 1–13, with a mean of 4.93 (SD = 2.67) and median of 5, while number of alive children had a range of 0–12, mean of 4.33 (SD = 2.40) and median of 4. The range of household size was 2–18, mean 7.39 (SD = 2.98) and median 7.

Almost 80% of the respondents were married and 63% reported housewife as their occupation, while approximately half (52%) had no education. Husband was reported as the main income provider of the household by 75% of respondents. Demographic characteristics are reported in Table 1.

**Table 1:** Demographic characteristics of participants

Characteristic	n (%)
Age (*N* = 789)	
*= < 27*	410 (52.0)
*28* +	379 (48.0)
Marital status (*N* = 789)	
*Married*	605 (76.7)
*Divorced/widowed/separated*	184 (23.3)
Education level (*N* = 786)	
*No education*	412 (52.4)
*Primary*	235 (29.9)
*Secondary* +	139 (17.7)
Education level of husband (*N* = 761)	
*No education*	283 (37.2)
*Primary*	177 (23.3)
*Secondary* +	301 (39.6)
Occupation (*N* = 789)	
*Housewife*	495 (62.7)
*Farmer*	82 (10.4)
*Employee*	96 (12.2)
*Business*	82 (10.4)
*Other*	34 (4.3)
Husband occupation (*N* = 752)	
*Unemployed*	114 (15.2)
*Farmer*	111 (14.8)
*Businessman*	261 (34.7)
*Employee*	162 (21.5)
*Other*	104 (13.8)
Breadwinner (*N* = 769)	
*Yourself*	128 (16.6)
*Your husband*	580 (75.4)
*Other*	61 (7.9)
Household size (*N* = 786)	
= < 7	438 (55.7)
8 +	348 (44.3)

#### ANC coverage and other pregnancy related behaviour and characteristics

About 85% of interviewed women had attended at least one ANC visit, while 23% had attended at least four ANC visits, indicating a 73% drop-out rate between the first and fourth visit. Slightly over half of respondents (51%) reported having given birth in a health facility during their last pregnancy. Almost 95% of respondents reported having ever started breastfeeding their new-born child. The attitude of husband/family towards ANC attendance was reported as “positive” by 90% of respondents, and 85% had been encouraged to attend ANC. Almost 60% reported having ever experienced a problem during a pregnancy. ANC coverage and other pregnancy related behaviour and characteristics are reported in Table 2.

**Table 2:** ANC coverage and other pregnancy related behaviour and characteristics

Characteristic	n (%)
Number of ANC visits (*N* = 792)	
*None/I did not attend any ANC*	119 (15.0)
*One*	157 (19.8)
*Two*	178 (22.5)
*Three*	156 (19.7)
*Four* +	182 (23.0)
At least one ANC visit (*N* = 792)	
*Yes*	673 (85.0)
*No*	119 (15.0)
At least 4 ANC visits (*N* = 792)	
*Yes*	182 (23.0)
*No*	610 (77.0)
Place of delivery (*N* = 786)	
*Home (FCHW/TBA)*	383 (48.7)
*Health facility (including private)*	403 (51.3)
Breastfeeding (*N* = 789)	
*Yes*	748 (94.8)
*No*	41 (5.2)
Total children (*N* = 790)	
*1–3*	287 (36.3)
*4–7*	354 (44.8)
*8* +	149 (18.9)
Alive children (*N* = 788)	
= < *3*	343 (43.5)
*4–7*	358 (45.4)
*8* +	87 (11.0)
Distance to health facility (*N* = 757)	
*Not a problem*	311 (41.1)
*Average*	339 (44.8)
*Big problem*	107 (14.1)
Cost to access health services (*N* = 766)	
*Not a problem*	311 (40.6)
*Average*	346 (45.2)
*Big problem*	109 (14.2)
Husband/family attitude towards ANC attendance (*N* = 756)	
*Positive*	681 (90.1)
*Negative*	75 (9.9)
Encouragement to attend ANC (*N* = 774)	
*Yes*	659 (85.1)
*No*	115 (14.9)
Midwife in health facility (*N* = 770)	
*Yes*	561 (72.9)
*No*	114 (14.8)
*I don’t know*	95 (12.3)
Knowledge of complications (*N* = 770)	
*Yes*	681 (88.4)
*No*	89 (11.6)
Problem during past pregnancy (*N* = 764)	
*Yes*	458 (60.0)
*No*	256 (33.5)
*Don’t remember*	50 (6.5)

### Factors associated with utilization of at least 1 ANC consultation

The odds of attending at least 1 ANC consultation were 13 times higher when the woman had been encouraged to attend ANC (crude OR 13.47, 95%CI 8.46–21.43). After adjusting for all other variables, the odds ratio was still 8.22 (95%CI 4.36–15.49). Negative attitude of a husband/partner/family was associated with 67% lower odds of attending at least 1 ANC consultation (adjusted OR 0.33, 95%CI 0.16–0.69).

Compared to women without formal education, having completed primary or higher education was associated with increased odds of attending at least 1 ANC consultation: crude OR 2.19 (95%CI 1.33–3.59) and 1.96 (95%CI 1.09–3.54) for primary or higher education, respectively; however, the association was no longer significant after adjusting for other variables. Similarly, knowledge of complications appeared to be associated with ANC attendance (crude OR 1.86, 95%CI 1.08–3.20), but after adjusting for other variables, the association was no longer significant. Not knowing whether there is a midwife in the facility decreased the odds of attending at least 1 ANC consultation by 58% (adjusted OR 0.42, 95%CI 0.17–1.04).

Crude and adjusted association between exposure variables and at least 1 ANC consultation are reported in Table 3.

**Table 3:** Crude and adjusted association between exposure variables and at least 1 ANC visit

	**Crude**		**Adjusted**	
**Variable**	**Crude OR (95% CI)**	*P*-value*	**Adjusted OR (95% CI)**^**a**^	*P*-value*
Age				
* = < 27*	1			
*28* +	0.71 (0.48–1.06)	0.097		
Marital status				
*Married*	1			
*Divorced/widowed/separated*	0.65 (0.42–1.01)	0.054		
Education level				
*No education*	1		1	
*Primary*	2.19 (1.33–3.59)	0.002	1.31 (0.64–2.71)	0.461
*Secondary* +	1.96 (1.09–3.54)	0.025	1.11 (0.46–2.67)	0.818
Partner’s education level				
*No education*	1		1	
*Primary*	1.74 (1.02–2.96)	0.042	1.03 (0.47–2.25)	0.951
*Secondary* +	1.71 (1.09–2.67)	0.019	0.88 (0.42–1.86)	0.744
Occupation				
*Housewife*	1			
*Farmer*	1.01 (0.52–1.95)	0.979		
*Employee*	0.93 (0.51–1.71)	0.825		
*Business*	0.92 (0.48–1.75)	0.795		
*Other*	1.30 (0.44–3.79)	0.634		
Partner’s occupation				
*Unemployed*	1			
*Farmer*	0.84 (0.40–1.77)	0.643		
*Businessman*	1.65 (0.82–3.30)	0.161		
*Employee*	0.62 (0.32–1.20)	0.154		
*Other*	0.41 (0.21–0.82	0.012		
Total children				
*1–3*	1			
*4–7*	0.76 (0.48–1.18)	0.217		
*8* +	0.81 (0.46–1.42)	0.465		
Alive children				
= < *3*	1			
*4–7*	0.89 (0.59–1.36)	0.603		
*8* +	0.70 (0.38–1.31)	0.271		
Household size				
= < *7*	1			
*8* +	0.88 (0.59–1.30)	0.519		
Breadwinner				
*Yourself*	1		1	
*Your husband/partner*	2.10 (1.30–3.40)	0.003	1.72 (0.84–3.54)	0.139
*Other*	0.98 (0.48–2.03)	0.964	0.70 (0.25–1.98)	0.506
Distance to health facility				
*Not a problem*	1		1	
*Average*	1.10 (0.66–1.83)	0.712	0.85 (0.41–1.75)	0.139
*Big problem*	0.18 (0.11–0.31)	< 0.001	0.70 (0.25–1.98)	0.506
Cost to access health services				
*Not a problem*	1			
*Average*	1.13 (0.69–1.84)			
*Big problem*	0.23 (0.14–0.40)			
Husband/partner/family attitude towards ANC attendance				
*Positive*	1		1	
*Negative*	0.15 (0.09–0.25)	< 0.001	0.33 (0.16–0.69)	0.003
Encouragement to attend ANC				
*No*	1		1	
*Yes*	13.47 (8.46–21.43)	< 0.001	8.22 (4.36–15.49)	< 0.001
Midwife in health facility				
*No*	1		1	
*Yes*	2.00 (1.13–3.54)	0.017	1.55 (0.69–3.49)	0.286
*I don’t know*	0.29 (0.15–0.54)	< 0.001	0.42 (0.17–1.04)	0.060
Knowledge of complications				
*No*	1		1	
*Yes*	1.86 (1.08–3.20)	0.026	1.32 (0.59–2.96)	0.507
Problem during past pregnancy				
*No*	1			
*Yes*	1.08 (0.70–1.67)	0.734		
*Don’t remember*	0.48 (0.23–0.99)	0.047		

^*^ *P*-value from Wald Test

^a^ Adjusted for education level of respondent and husband, breadwinner, distance to health facility, cost to access health services, husband/family attitude towards ANC attendance, encouragement to attend ANC, midwife in health facility and knowledge of complications

### Factors associated with completion of the minimum cycle of ANC consultations (4 +)

Unlike with attending at least 1 ANC consultation, where we found no association with occupation, being a housewife increased the odds of attending at least 4 ANC consultations compared to other occupation (Table 4). However, women working in business had about 60% lower odds of completing at least four ANC consultations (adjusted OR 0.41, 95%CI 0.19–0.90). Similarly, odds decreased by approximately half if someone else was the breadwinner of the household compared to the woman herself (husband: adjusted OR 0.55, 95%CI 0.31–0.99; other: adjusted OR 0.34, 95%CI 0.13–0.89). Divorced/widowed/separated women also had lower odds of attending at least four ANC consultations (adjusted OR 0.53, 95%CI 0.30–0.92).

Encouragement to attend ANC was again associated with increased attendance, with adjusted OR 2.47 (95%CI 1.16–5.23). Negative attitude of husband/family towards ANC attendance decreased the odds by 69% (crude OR 0.31, 95%CI 0.14–0.69), however the association was no longer significant after adjusting for other variables.

Knowing whether there is a midwife in the facility increased the odds by 87% (adjusted OR 1.87, 95%CI 1.03–3.41). Knowledge of complications or having experienced problem during past pregnancies was not found to be associated with attending at least 4 ANC consultations. Crude and adjusted associations between exposure variables and at least 4 ANC consultations are presented in Table 4.

**Table 4:** Crude and adjusted association between exposure variables and at least 4 ANC visits

	**Crude**		**Adjusted**	
**Variable**	**Crude OR (95% CI)**	*P*-value*	**Adjusted OR (95% CI)**^**a**^	*P*-value*
Age				
* = < 27*	1		1	
*28* +	1.56 (1.12–2.18)	0.009	1.25 (0.82–1.91)	0.303
Marital status				
*Married*	1		1	
*Divorced/widowed/separated*	0.53 (0.34–0.82)	0.004	0.53 (0.30–0.92)	0.024
Education level				
*No education*	1			
*Primary*	0.64 (0.43–0.95)	0.027		
*Secondary* +	0.82 (0.52–1.29)	0.388		
Partner’s education level				
*No education*	1			
*Primary*	0.67 (0.42–1.07)	0.096		
*Secondary* +	0.99 (0.68–1.45)	0.966		
Occupation				
*Housewife*	1		1	
*Farmer*	0.42 (0.22–0.82)	0.011	0.50 (0.24–1.03)	0.059
*Employee*	0.63 (0.36–1.09)	0.097	0.65 (0.34–1.25)	0.195
*Business*	0.47 (0.25–0.89)	0.020	0.41 (0.19–0.90)	0.026
*Other*	0.71 (0.30–1.66)	0.424	0.70 (0.26–1.91)	0.485
Partner’s occupation				
*Unemployed*	1			
*Farmer*	0.77 (0.41–1.43)	0.4022		
*Businessman*	1.01 (0.61–1.68)	0.962		
*Employee*	0.90 (0.51–1.57)	0.705		
*Other*	0.66 (0.34–1.26)	0.204		
Total children				
*1–3*	1			
*4–7*	1.29 (0.88–1.90)	0.190		
*8* +	1.77 (1.12–2.80)	0.015		
Alive children				
= < *3*	1			
*4–7*	1.16 (0.81–1.65)	0.427		
*8* +	1.82 (1.09–3.06)	0.024		
Household size				
= < *7*	1		1	
*8* +	1.63 (1.17–2.27)	0.004	1.51 (0.98–2.31)	0.060
Breadwinner				
*Yourself*	1		1	
*Your husband/partner*	0.97 (0.62–1.51)	0.886	0.55 (0.31–0.99)	0.045
*Other*	0.54 (0.24–1.22)	0.140	0.34 (0.13–0.89)	0.029
Distance to health facility				
*Not a problem*	1		1	
*Average*	0.42 (0.29–0.61)	< 0.001	0.44 (0.29–0.66)	< 0.001
*Big problem*	0.34 (0.19–0.61)	< 0.001	0.57 (0.29–1.11)	0.101
Cost to access health services				
*Not a problem*	1			
*Average*	0.68 (0.48–0.97)	0.033		
*Big problem*	0.39 (0.22–0.71)	0.002		
Husband/partner/family attitude towards ANC attendance				
*Positive*	1		1	
*Negative*	0.31 (0.14–0.69)	0.004	0.55 (0.23–1.31)	0.177
Encouragement to attend ANC				
*No*	1		1	
*Yes*	2.71 (1.49–4.95)	0.001	2.47 (1.16–5.23)	0.019
Midwife in health facility				
*No*	1		1	
*Yes*	1.81 (1.07–3.06)	0.028	1.87 (1.03–3.41)	0.040
*I don’t know*	0.72 (0.33–1.58)	0.415	1.01 (0.40–2.52)	0.986
Knowledge of complications				
*No*	1			
*Yes*	1.45 (0.82–2.56)	0.203		
Problem during past pregnancy				
*No*	1			
*Yes*	0.86 (0.60–1.23)	0.419		
*Don’t remember*	0.48 (0.21–1.12)	0.088		

^*^P-value from Wald Test

^a^ Adjusted for age, marital status, occupation, household size, breadwinner, distance to health facility, husband/family attitude towards ANC attendance, encouragement to attend ANC and midwife in health facility

### Association between ANC access and delivery in a health structure

Access to at least 1 ANC visit increased the odds of delivering in a health structure by 2.65-fold (crude OR 2.65, 95%CI 1.75–4.03), however, after adjusting for potential confounders, the association was no longer significant (adjusted OR 1.43, 95%CI 0.81–2.52). Access to at least 4 ANC visits increased the odds by 50% even after adjusting (adjusted OR 1.50, 95%CI 1.01–2.24).

High education level (secondary or higher) of both the woman and her husband increased the odds of delivering in a health structure compared to having no education (adjusted OR: 1.79, 95%CI 1.09–2.94 and 1.95, 95%CI 1.29–2.97, respectively).

Not knowing whether there is a midwife in the health facility decreased the odds of delivering in a health structure by 72% (adjusted OR 0.28, 95%CI 0.14–0.57). When cost to access health services was reported as “big problem”, odds of delivering in a health structure were also 40% lower (crude OR 0.60, 95%CI 0.38–0.94); however, the association was no longer significant after adjusting for other variables.

Crude and adjusted associations between exposure variables and place of delivery are presented in Table 5.

**Table 5:** Crude and adjusted association between exposure variables and place of delivery

	**Crude**		**Adjusted**	
**Variable**	**Crude OR (95% CI)**	P-value*	**Adjusted OR (95% CI)**^**a**^	*P*-value*
Age				
* = < 27*	1			
*28* +	0.88 (0.67–1.17)	0.387		
Marital status				
*Married*	1			
*Divorced/widowed/separated*	1.06 (0.76–1.48)	0.730		
Education level				
*No education*	1		1	
*Primary*	1.46 (1.06–2.02)	0.021	1.14 (0.77–1.68)	0.527
*Secondary* +	2.94 (1.95–4.44)	< 0.001	1.79 (1.09–2.94)	0.021
Partner’s education level				
*No education*	1		1	
*Primary*	1.44 (0.99–2.11)	0.059	1.28 (0.83–1.97)	0.259
*Secondary* +	2.56 (1.83–3.57)	< 0.001	1.95 (1.29–2.97)	0.002
Occupation				
*Housewife*	1			
*Farmer*	0.44 (0.26–0.72)	0.001		
*Employee*	1.32 (0.85–2.07)	0.221		
*Business*	1.59 (0.98–2.59)	0.060		
*Other*	0.74 (0.37–1.49	0.398		
Husband’s occupation				
*Unemployed*	1			
*Farmer*	0.66 (0.38–1.12)	0.124		
*Businessman*	1.50 (0.96–2.33)	0.075		
*Employee*	1.32 (0.81–2.13)	0.265		
*Other*	1.27 (0.75–2.17)	0.379		
Total children				
*1–3*	1			
*4–7*	1.02 (0.75–1.40)	0.884		
*8* +	0.66 (0.44–0.98)	0.040		
Alive children				
= < *3*	1			
*4–7*	1.07 (0.80–1.44)	0.643		
*8* +	0.63 (0.39–1.01)	0.056		
Household size				
= < *7*	1			
*8* +	1.08 (0.81–1.43)	0.602		
Breadwinner				
*Yourself*	1			
*Your husband*	0.89 (0.61–1.32)	0.572		
*Other*	0.60 (0.32–1.12)	0.107		
Distance to health facility				
*Not a problem*	1		1	
*Average*	1.25 (0.92–1.71)	0.154	1.49 (0.99–2.26)	0.058
*Big problem*	0.64 (0.40–1.00)	0.051	1.45 (0.70–2.99)	0.316
Cost to access health services				
*Not a problem*	1		1	
*Average*	0.95 (0.70–1.29)	0.733	0.75 (0.50–1.13)	0.166
*Big problem*	0.60 (0.38–0.94)	0.025	0.87 (0.43–1.77)	0.708
Husband/family attitude towards ANC attendance				
*Positive*	1			
*Negative*	0.84 (0.52–1.36)	0.480		
Encouragement to attend ANC				
*No*	1		1	
*Yes*	2.54 (1.66–3.87)	< 0.001	1.36 (0.78–2.40)	0.280
Midwife in health facility				
*No*	1		1	
*Yes*	1.41 (0.94–2.11)	0.095	1.44 (0.91–2.25)	0.116
*I don’t know*	0.27 (0.14–0.51)	< 0.001	0.28 (0.14–0.57)	< 0.001
Knowledge of complications				
*No*	1			
*Yes*	0.89 (0.57–1.38)	0.589		
Problem during past pregnancy				
*No*	1			
*Yes*	1.10 (0.81–1.49)	0.560		
*Don’t remember*	0.79 (0.43–1.46)	0.454		
Access to at least 1 ANC visit				
*No*	1		1	
*Yes*	2.65 (1.75–4.03)	< 0.001	1.43 (0.81–2.52)	0.220
Access to at least 4 ANC visits				
*No*	1		1	
*Yes*	1.53 (1.09–2.14)	0.013	1.50 (1.01–2.24)	0.043

^*^P-value from Wald Test

^a^Adjusted for education level of respondent and husband, distance to health facility, cost to access health services, encouragement to attend ANC, midwife in health facility, access to at least 1 ANC visit and access to at least 4 ANC visits

### Association between ANC access (and other variables) and initiation of breastfeeding

Access to at least 1 ANC consultation was associated with 2.69 times higher odds of initiating breastfeeding (95%CI 1.07–6.74). However, no evidence of association was found with access to at least 4 ANC consultations (crude OR 1.24, 95%CI 0.56–2.74).

Education of both the woman and her husband increased the odds of initiating breastfeeding. Compared to no education for the respondent, having completed primary school was associated with 2.65 times higher odds of initiating breastfeeding (95%CI 0.95–7.33). Husband’s primary education also increased the odds of initiating breastfeeding by 2.5-fold; however, the association was no longer significant after adjusting for other variables.

Husband’s negative attitude towards ANC attendance decreased the odds of initiating breastfeeding by 64% (crude OR 0.36, 95%CI 0.17–0.79); however, the association was not significant after adjusting for other variables.

See Table 6 for crude and adjusted association between explore variables and starting breastfeeding for a newborn.

**Table 6:** Crude and adjusted association between exposure variables and starting breastfeeding for newborn

	**Crude**		**Adjusted**	
**Variable**	**Crude OR (95% CI)**	*P*-value*	**Adjusted OR (95% CI)**^**a**^	*P*-value*
Age				
* = < 27*	1			
*28* +	1.26 (0.66–2.47)	0.469		
Marital status				
*Married*	1		1	
*Divorced/widowed/separated*	0.57 (0.29–1.10)	0.095	0.54 (0.25–1.14)	0.107
Education level				
*No education*	1		1	
*Primary*	2.87 (1.17–7.02)	0.021	2.65 (0.95–7.33)	0.061
*Secondary* +	2.56 (0.88–7.42)	0.083	2.32 (0.71–7.65)	0.165
Husband’s education level				
*No education*	1		1	
*Primary*	2.51 (1.00–6.28)	0.050	2.19 (0.79–6.11)	0.133
*Secondary* +	2.12 (1.03–4.34)	0.041	1.37 (0.59–3.22)	0.465
Occupation				
*Housewife*	1			
*Farmer*	1.52 (0.45–5.14)	0.498		
*Employee*	5.43 (0.73–40.49)	0.099		
*Business*	0.62 (0.26–1.47)	0.279		
*Other*	0.90 (0.20–3.94)	0.885		
Husband’s occupation				
*Unemployed*	1			
*Farmer*	4.15 (0.86–20.01)	0.076		
*Businessman*	1.33 (0.54–3.27)	0.530		
*Employee*	1.47 (0.53–4.03)	0.458		
*Other*	0.91 (0.33–2.53)	0.863		
Total children				
*1–3*	1			
*4–7*	1.65 (0.79–3.46)	0.183		
*8* +	0.87 (0.39–1.96)	0.740		
Alive children				
= < *3*	1			
*4–7*	1.05 (0.53–2.05)	0.895		
*8* +	1.14 (0.38–3.46)	0.818		
Household size				
= < *7*	1			
*8* +	0.96 (0.51–1.82)	0.902		
Breadwinner				
*Yourself*	1			
*Your husband*	1.23 (0.52–2.90)	0.638		
*Other*	0.64 (0.19–2.09)	0.457		
Distance to health facility				
*Not a problem*	1			
*Average*	1.10 (0.54–2.24)	0.788		
*Big problem*	0.91 (0.35–2.39)	0.848		
Cost to access health services				
*Not a problem*	1			
*Average*	1.48 (0.71–3.10)	0.297		
*Big problem*	0.57 (0.25–1.28)	0.174		
Husband/partner/family attitude towards ANC attendance				
*Positive*	1		1	
*Negative*	0.36 (0.17–0.79)	0.011	0.66 (0.26–1.67)	0.385
Encouragement to attend ANC				
*No*	1		1	
*Yes*	1.58 (0.70–3.53)	0.268	0.64 (0.23–1.81)	0.404
Midwife in health facility				
*No*	1			
*Yes*	0.73 (0.28–1.92)	0.526		
*I don’t know*	2.13 (0.40–11.25)	0.372		
Knowledge of complications				
*No*	1			
*Yes*	1.17 (0.44–3.07)	0.754		
Problem during past pregnancy				
*No*	1			
*Yes*	1.09 (0.55–2.16)	0.801		
*Don’t remember*	0.91 (0.25–3.28)	0.881		
Access to at least 1 ANC visit				
*No*	1		1	
*Yes*	3.18 (1.61–6.25)	0.001	2.69 (1.07–6.74)	0.035
Access to at least 4 ANC visits				
*No*	1			
*Yes*	1.24 (0.56–2.74)	0.592		
Place of delivery				
*Home (FCHW/TBA)*	1			
*Health facility (include private)*	0.76 (0.40–1.45)	0.411		

^*^P-value from Wald Test

^a^ Adjusted for marital status, education level of respondent and husband, husband/family attitude towards ANC attendance, encouragement to attend ANC and access to at least 1 ANC visit

### Exploratory questions on access and beliefs

Long distance was the most common reason not to attend ANC (56%). Not seeing the need to attend ANC was reported by 18% of respondents and cost of care by 13%. Almost a fifth of all women reported not having received any information about ANC, while a health institution and relatives were the two most commonly reported sources of information with 31% and 27%, respectively.

Women who reported having been encouraged to attend ANC were asked who the source of encouragement was. Husband was mentioned by 46% and mother-in-law by 15% of respondents, while 32% of women mentioned a health worker.

Almost two thirds (61%) responded being aware of benefits of ANC attendance for both maternal and child health. When asked about complications, 61% of the women reported being aware of anaemia, while some of the more severe complications such as hypertension, malposition of the foetus and obstructed/prolonged labour were known by 9–12%. 28% of women had experienced haemorrhage in their last pregnancy, and 16% had experienced either abortion or stillbirth. 40% had been performed episiotomy, 25% assisted vaginal delivery and 22% perennial suture/re-infibulation/reparation.

Reason to choose the place of delivery was reported as “close to where I live” by 41% of the respondents, followed by “little or no expenses” with 29%. Factors directly related to the service (good behaviour of health workers, convenient time of services and high-quality services) were mentioned by 4–12% of women.

Responses to exploratory questions on access and beliefs are listed in Table 7.

**Table 7:** Exploratory questions on access and beliefs

	**n (%)**
Reason for not attending ANC (*N* = 309)^a^	
*Long distance from home to the clinic*	174 (56.31)
*Cost of care*	41 (13.27)
*Feeling good health/ don’t see the necessity to attend ANC*	57 (18.45)
*Too busy with other children / housework*	29 (9.39)
*Long waiting time*	23 (7.44)
*Husband disapproval / family*	11 (3.56)
*Poor quality service*	13 (4.21)
*I prefer the TBA service*	8 (2.59)
*Clinic operating hours*	3 (0.97)
*Taboos of the drugs during pregnancy*	0 (0)
*Others*	12 (3.88)
Source of information on ANC services (*N* = 769)	
*I did not get any information*	139 (18.08)
*Health institution / midwife*	240 (31.21)
*FCHW/TBA*	132 (17.17)
*Radio*	78 (10.14)
*Social media*	52 (6.76)
*Relatives*	205 (26.66)
*Others*	66 (8.58)
Source of encouragement (*N*=665)^b^	
*Husband/partner*	307 (46.17)
*Mother-in-law*	101 (15.19)
*Friends*	131 (19.70)
*Health workers*	211 (31.73)
*Others*	64 (9.62)
Maternal services available at nearest health facility (according to respondent) (*N* = 763)	
*ANC*	473 (61.99)
*Delivery*	352 (46.13)
*Family planning*	121 (15.86)
*Curative service*	339 (44.43)
*Immunization*	435 (57.01)
*Others*	82 (10.75)
Known benefits of ANC attendance (*N* = 765)	
*Maternal health*	222 (29.02)
*Child health*	107 (13.99)
*Both*	468 (61.18)
*Don’t know*	67 (8.76)
*Others*	26 (3.40)
Knowledge of complications (*N* = 681)^c^	
*Persistent vomiting*	357 (52.42)
*Anemia*	413 (60.65)
*Swelling of lower limbs*	241 (35.39)
*Headache*	282 (41.41)
*Vaginal bleeding*	148 (21.73)
*Hypertension*	64 (9.40)
*Malposition of the fetus*	67 (9.84)
*Obstructed/prolonged labour*	82 (12.04)
*Retained placenta*	35 (5.14)
Problems during last pregnancy (*N* = 439)^d^	
*Anemia*	262 (59.68)
*Abortion*	70 (15.95)
*Still birth*	70 (15.95)
*Malaria*	67 (15.26)
*Hemorrhage*	122 (27.79)
*STI*	46 (10.48)
*Others*	47 (10.71)
Procedures performed (*N* = 747)	
*Episiotomy*	298 (39.89)
*Caesarian Section*	83 (11.11)
*Assisted Vaginal Delivery*	187 (25.03)
*Suture/re-infibulation/perennial reparation*	161 (21.55)
*Others*	124 (16.60)
Reason to choose place of delivery (*N* = 786)	
*Close to where I live*	325 (41.35)
*Little or no expenses*	225 (28.63)
*Privacy / confidentiality*	157 (19.97)
*Family support*	92 (11.70)
*Good behavior of the health workers*	97 (12.34)
*Preparation of birth (well explained by MW)*	65 (8.27)
*Convenient time of services*	37 (4.71)
*High quality services*	82 (10.43)
*Others*	20 (2.54)
Recommendations to improve services in health facility (*N* = 774)	
*Increase number of health workers*	198 (25.58)
*Improve availability of drugs and supplies*	277 (35.79)
*Increase working hours*	206 (26.61)
*Health workers should respect the clients*	164 (21.19)
*Availability of means of transportation*	273 (35.27)
*Others*	93 (12.02)

^a^Includes those who reported less than 4 ANC visits

^b^ Includes those who reported having been encouraged to attend ANC

^c^ Includes those who reported knowing complications

^d^ Includes those who reported a problem in last pregnancy

## Discussion

### Summary of results and comparison with other studies

In our study, we found that more than four out of five women had attended at least one antenatal (ANC) consultation, while only one out of five women had completed the minimum cycle of at least four ANC consultations. The coverage of at least one ANC consultation was well beyond the official figure in Somalia, which was estimated as 32% in 2020 by the Directorate of National Statistics [13]. While coverage of ANC1, as revealed by this survey, was slightly lower than the one estimated by the ICRC/SRCS routine data monitoring (85% vs 100%, respectively), we found a very large difference between the coverage of ANC4 estimated by this survey (23%) versus the one estimated by routine data (88%). Such difference needs to be further investigated, but it is likely to be due to wrong estimation of denominators or issues with reporting.

Encouragement to attend ANC was the factor most strongly associated with attending ANC, and the association was stronger when looking at attending at least one ANC consultation. Most often, encouragement came from husband or health workers. Negative attitude of husband, partner or family was also a predictor of lower attendance of at least one visit. Other studies found similar relation between the encouragement or support given to the woman and tendency to seek ANC care [20, 21]. The association between negative attitude of family and ANC attendance has also been reported elsewhere [22, 23]. This finding reflects the decision-making role of husband and family in Somalia, including for matters regarding maternal health. Community participation in enhancing the use of health services including ANC is a key point, while a negative attitude of husband or family members can be an unbearable barrier to access ANC.

We found that women without formal education were less likely to attend ANC consultation compared to women having completed primary or higher education. This goes along with other studies conducted in African countries where level of education for women or both women and husbands has been shown to positively influence attendance to ANC [24–26]. The Voluntary National Review Report 2022 also mentions the level of education as one of the strong determining factors for receiving ANC from skilled health care providers in Somalia [14].

Unlike with attending at least one ANC consultation, where we found no association between women’s occupation and attendance to ANC, we saw that being a housewife increased the chances of attending at least four ANC consultations compared to other occupations. In contrast to our findings, studies in Papua New Guinea and Benin have suggested that working women have higher odds of early initiation and completion of at least 4 ANC visits [27, 28].

Not knowing whether there is a midwife in the health facility decreased the odds of attending at least one ANC visit by more than half, while knowing that there is a midwife increased the odds of at least four visits. Human resources for health are an important pillar to enable provision of health care. However, availability of adequate human resources including midwives is not adequate on its own, rather, it needs to be accompanied by awareness raising on the available services at the health facility. Furthermore, a previous study from Somaliland indicated that poor attitude of staff can negatively influence women’s utilization of maternal and child health services [29].

Few reported cost of care as a reason not to attend ANC as such, but if cost was reported as a “big problem”, odds of attending ANC decreased by more than half. Free health service in the study context could partly explain this. Various studies have reported cost to be a barrier to access health services [30–32]. While we found no association with the attendance itself, long distance from home to the clinic was the most often reported reason for not attending ANC.

Approximately half of the women had delivered in a health facility. We found ANC attendance likely to be associated with delivering in a health structure. Similar studies found that ANC visits are an essential and significant determinant of delivery at a health facility [33, 34].

High education level of both the woman and her husband increased the odds of delivering in a health structure. In addition, education has the potential to empower women and increase their health literacy. Factors decreasing the odds of a health structure delivery included not knowing whether there is a midwife and reporting cost to access health services as a “big problem”.

Attending at least one ANC visit increased the odds of initiating breastfeeding, while we found no association for the minimum cycle of at least four visits. This highlights the importance of at least starting ANC, since this first contact with the health services is an important moment to deliver health promotion messages. Furthermore, delivering in a health structure and being breastfed can be gamechangers for any child in a country with high malnutrition rates, while good health is also an enabler of education later in life. In our study 95% of the respondent mothers reported that they introduced breastfeeding within one hour of childbirth. This is in line with the results of the Somali demographic survey conducted in 2020 [13] and also supported by the findings of other studies done in Asia and Africa [35–38].

### Strengths and limitations

A major strength of the study is that we used a validated cluster random sampling method developed by WHO [19], which ensured we had a representative sample of the population of interest. Volunteers who conducted the interviews were trained on the methodology to select women in each village, minimizing selection bias. We also achieved a very high response rate, which allowed us to reach the pre-determined sample size and minimize selection bias. Finally, the choice to include a large variety of questions in our survey meant that it was possible to explore associations between multiple factors.

While we achieved the required sample size, it seems we might not have had sufficient power to show some associations, especially after adjusting for several variables. Therefore, it remains unknown whether some factors were associated with ANC attendance or not. Some variables also had a considerable number of missing values. This could cause some bias due to nondifferential or differential misclassification, however, we do not know the direction of this misclassification. Since we chose to focus on having a representative sample of the South-Central regions of Somalia as a whole, we cannot make inferences nor make recommendations for any specific geographical area. Lastly, we cannot rule out the possibility of residual confounding in spite of the analysis strategy.

### Public health implications and recommendations

#### Community health education

In Somalia, husbands play a key role in decision making for women, hence there is a need to increasingly involve men in health education programmes that aim at promoting the effective utilization of antenatal care services. Men can also have an important role in promoting other preventive services for their families, beyond ANC, such as family planning, post-natal care and childhood vaccination.

Women are to be encouraged to seek antenatal care, deliver at a health facility and initiate breastfeeding. However, for optimal results, these messages will need to be communicated across communities. Ensuring access to even the most basic information about available services and their importance can make a difference for ANC attendance in Somalia, and this is likely to be generalisable to other healthcare as well.

#### Integrated mobile outreach services

Since long distance from home to the clinic was the most often reported reason for not attending ANC, it will be important to provide outreach services for the women who have more difficulties to access health services and finish four antenatal visits. The outreach, organized by the health facility itself, services can also reduce the burden of transportation costs for the mothers.

#### Capacity building of midwives

As knowing that there is a midwife in the facility increased the odds of seeking ANC, and other studies have suggested that unprofessional behaviour of facility personnel can influence health seeking behaviour during pregnancy, it will be important for the midwives to be trained on the different components of care to ensure these matters do not affect women’s access to quality services.

#### Proposed future research

Similar studies need to be conducted in other conflict-affected countries in order to explore factors associated with sexual & reproductive health services and address the possible obstacles.

Specifically, for Somalia, qualitative designs may be considered to explore in-depth reasons for non-attendance starting with the factors that were highly associated in this study. In addition, exploring the reasons for different ANC coverage estimation between routine and survey data is recommended.

In order to assess which interventions may increase access to services (e.g., cash vouchers or similar), randomized-controlled trials can be conducted.

## Conclusions

In our study, receiving encouragement was the single most important factor increasing women’s ANC utilization in Somalia, while negative attitude of husband or family had the opposite effect. Knowing there is a midwife also increased the attendance to ANC. On the other hand, long distance was the most common reason not to attend ANC. These results highlight that family and especially husband have a strong influence in women’s ANC attendance in Somalia, which can in turn increase the likelihood of delivering in a health facility and initiating breastfeeding, contributing to maternal and child health more broadly. Women and their families need to have access to information about the benefits and the availability of services, and potential solutions to this need can include community health education and integrated mobile outreach services, where men can also have an active role. Qualitative research to explore in-depth reasons for non-attendance and randomized controlled trials to assess potential interventions could be conducted to further explore this topic.

## Data Availability

The datasets used and/or analyzed during the study are available from the corresponding author on reasonable request.
